# Influence of pCP1NetB ancillary genes on the virulence of *Clostridium perfringens* poultry necrotic enteritis strain CP1

**DOI:** 10.1186/s13099-016-0152-y

**Published:** 2017-01-21

**Authors:** Hongzhuan Zhou, Dion Lepp, Yanlong Pei, Mei Liu, Xianhua Yin, Rongcai Ma, John F. Prescott, Joshua Gong

**Affiliations:** 1 Beijing Agro-Biotechnology Research Center, Beijing Academy of Agriculture and Forestry Sciences, Beijing, 100097 China; 2 Guelph Research and Development Centre, Agriculture and Agri-Food Canada, 93 Stone Road West, Guelph, ON N1G 5C9 Canada; 3 Department of Pathobiology, University of Guelph, Guelph, ON N1G 2W1 Canada

**Keywords:** Necrotic enteritis, *Clostridium perfringens*, Plasmid-deficient mutant, NELoc-1, NetB

## Abstract

**Background:**

Necrotic enteritis (NE) is an economically important disease of poultry caused by certain *Clostridium perfringens* type A strains. The NetB toxin plays a critical role in the pathogenesis of NE. We previously demonstrated that *netB* is located within a 42 kb plasmid-encoded pathogenicity locus (NELoc-1), which also encodes 36 additional genes. Although NetB clearly plays a role in pathogenesis, the involvement of the other NELoc-1 genes has not yet been established. The current study was to provide experimental evidence to confirm the involvement of these genes in NE pathogenesis.

**Results:**

The present study has characterized a virulent *C. perfringens* strain (CP1) that has spontaneously lost the NELoc-1-encoding plasmid, pCP1netB. When assessed for cytotoxicity on Leghorn Male Hepatoma (LMH) cells, the culture supernatant of the pCP1netB-deficient CP1 variant (CP1ΔpCP1netB) demonstrated significantly reduced cytotoxicity compared to the wild-type. In addition, CP1ΔpCP1netB was unable to cause intestinal lesions in chickens in a NE disease model. When *netB* alone was introduced into CP1ΔpCP1netB, in vitro cytotoxicity was restored to the wild-type level; however, it did not completely restore virulence when used to challenge broiler chickens [mean lesion score of 0.71 compared to 3.23 in the wild type control group (n = 14)].

**Conclusions:**

The results of this study suggest that other genes present in NELoc-1, in addition to *netB*, are required for full virulence in the chicken challenge model.

## Background

Avian necrotic enteritis (NE) is caused by certain strains of type A *Clostridium perfringens*, an anaerobic Gram-positive bacterium. It is of significant economic importance to the poultry industry, estimated to cost approximately US$6 billion worldwide in 2015 [1]. The necrotic enteritis beta toxin-like (NetB) toxin was recently identified and shown to be a critical virulence factor in NE pathogenesis [2]. In these studies, a *netB* knockout mutant was unable to induce disease in an NE challenge chicken model system, while complementation with *netB* restored the virulence of the mutant [2]. Furthermore, several studies have examined *netB* prevalence in a wide variety of *C. perfringens* strains and found a strong correlation between the presence of *netB* and host disease status [3–6]. Recently, we took the approach of comparative genomics and identified a genetic “signature” of NE disease in chickens [7]. We found that *netB* resides on a 42 kb pathogenicity locus, NELoc-1, located on a plasmid (pNetB) and associated specifically with virulent strains. This locus contains 36 genes in addition to *netB*, several of which are predicted to play a role in virulence. Two other loci (NELoc-2 and -3) associated with NE-producing strains were also identified: NELoc-2 consists of 11 genes and is chromosomally-encoded, whereas NELoc-3 consists of five genes and is located on a plasmid (pCpb2) distinct from that harboring NELoc-1. The complete sequences of pNetB and pCpb2 from virulent strains originating in Canada and Australia have been reported and contain sequences with >99% identity to NELoc-1 and NELoc-3, respectively [8, 9]. The strict conservation of these loci suggests that they may encode genes important in the pathogenesis of NE. However, functional confirmation of these putative additional virulence factors has yet to be provided. The extrachromosomal location of most of the NE-associated genes (*i.e.* NELoc-1 and NELoc-3) presents an opportunity to remove these genes *en masse*, through plasmid loss, and thereby assess their collective role in virulence in a common genetic background. Furthermore, putative virulence genes may be reintroduced into the strains cured of their virulence plasmids, either individually or in combination, to assess each gene for phenotypic effects and possible interactions.

To extend our previous findings from the comparative genomic analyses, we have characterized a virulent *C. perfringens* strain (CP1) that has spontaneously lost its *netB*-carrying plasmid (pCP1netB). The virulence of the mutant, a *netB*-complemented derivative, and the wild-type strain has also been assessed both in vitro and in vivo. The results are reported herein.

## Methods

### Bacterial strains, media and growth conditions

*Clostridium perfringens* strain CP1 used in this study was a field isolate from an NE case in Ontario [10]. *C. perfringens* strains were routinely grown anaerobically at 37 °C in Brain Heart Infusion (BHI), Tryptone Proteose peptone Glucose (TPG), or Fluid Thioglycollate (FTG) broth (Difco). *C. perfringens* media were supplemented with 34 µg/ml chloramphenicol (Sigma-Aldrich, St. Louis, MO, USA) as required to retain the plasmid pJIR750::*netB* that carries a chloramphenicol resistance gene. *E. coli* Top 10 was used for cloning and grown at 37 °C in Luria–Bertani (LB) broth or agar (Difco), supplemented with 34 µg/ml chloramphenicol as required.

### Pulsed field gel electrophoresis (PFGE)

Pulsed field gel electrophoresis was performed on chromosomal and plasmid DNA as previously described [7]. DNA plugs for PFGE were prepared from overnight cultures of *C. perfringens* grown in BHI and the bacterial pellets incorporated into a final agarose concentration of 1% in PFGE-certified agarose (Bio-Rad, Hercules, CA, USA). Plugs were incubated overnight with gentle shaking at 37 °C in lysis buffer (0.5 M EDTA pH 8.0, 2.5% of 20% sarkosyl), 0.25% lysozyme (Sigma-Aldrich) and subsequently incubated in 2% proteinase K (Roche, Laval, QC, Canada) buffer for 2 days at 55 °C. Then plugs were equilibrated in 200 µl restriction buffer at room temperature for 20 min and digested with 10 U of *Not*I (New England Biolabs, Ipswich, MA) at 37 °C overnight. Electrophoresis was performed in a 1% PFGE-certified gel and separated with the CHEF-III PFGE system (Bio-Rad) in 0.5X Tris–borate-EDTA buffer at 14 °C at 6 V for 19 h with a ramped pulse time of 1–12 s. Gels were stained in ethidium bromide and visualized by UV light. Mid-Range II PFG markers (New England Biolabs) were used as a molecular DNA ladder.

### Genome sequencing and phylogenetic analysis

Total genomic DNA was isolated from strains CP1 and CP1ΔpCP1netB using the Gentra Puregene kit (Qiagen). Sequencing libraries were prepared with the Nextera XT DNA sample preparation kit (Illumina, San Diego, CA) according to the manufacturer’s instructions, using 1 ng of total gDNA as input. The library concentration was determined by quantitative PCR, and diluted to a final concentration of 8 ρM in 10 mM Tris–HCl, pH 8. Sequencing was performed on a MiSeq instrument (Illumina) using the 600v3 kit, to generate 300 bp paired-end reads. The quality of the resulting Fastq files was assessed with FastQC (http://www.bioinformatics.bbsrc.ac.uk/projects/fastqc), and reads were quality filtered and assembled with SPAdes v3.0 [11]. Phylogenetic trees based on whole genome sequence data were built using REALPHY [12], which uses Bowtie 2 [13] for mapping reads to a reference genome and RAxML [14] for tree-building. The ATCC13124 genome (gi_110798562) was used as a reference and *C. perfringens* strains ATCC3626 (gi_151558394), JGS1721 (gi_177911222), F262 (gi_422872590), NCTC8239 (gi_151558627), WAL-14572 (gi_373228597), JGS1987 (gi_151558496), JGS1495 (gi_151558295), Str.13 (gi_18308982), F4969 (gi_151558571), 1207_CPER (gi_875303967), JJC (gi_558869255), and CP4 (gi_942714078) were also included in the comparison.

### Construction of plasmid pJIR750::netB and complementation of CP1ΔpCP1netB

The *netB* complementation plasmid pJIR750::*netB* was constructed using primers CTC_netBF (CGGGATCCGTACCATTTAAATTAAGCAC) and CTC_netBR (TCGAGCTCCCCTCTATATACTATTGATTG), which contain *Bam*HI and *Sac*I restriction sites (underlined), respectively. They were used to amplify a 1551 bp fragment encompassing the wildtype *netB* gene and 300 bp of upstream sequence. Following digestion with *Bam*HI and *Sac*I, the fragment was purified with the Qiagen PCR purification kit (Qiagen, Mississauga, Ontario, Canada), and ligated into the *Bam*HI/*Sac*I sites of the *E. coli*-*C. perfringens* shuttle vector pJIR750 (a gift from Dr. Julian I. Rood, Monash University, Australia), which confers chloramphenicol resistance, and the resulting pJIR750::*netB* plasmid was confirmed by sequencing. pJIR750::*netB* was then introduced into CP1ΔpCP1netB by electroporation to generate CP1ΔpCP1netB(*netB* ^+^). The transformation mixture was plated onto BHI agar supplemented with 34 μg/ml chloramphenicol and incubated overnight at 37 °C under anaerobic conditions. The CP1ΔpCP1netB(*netB* +) transformants were confirmed by PCR amplification of a 302 bp fragment using primers QnetBF (AGTGTAATTAGTACAAGCC) and QnetBR (GGCCATTTCATTTTTCCGTAA) and the following cycling conditions: initial denaturation at 94 °C for 5 min; 30 cycles at 94 °C for 30 s, 47 °C for 30 s and 68 °C for 25 s; final extension at 68 °C for 10 min.

### LMH cytotoxicity assay

Lactate dehydrogenase (LDH) cytotoxicity assays were performed essentially as described [15, 16]. Briefly, chicken Leghorn male hepatoma (LMH) cells (ATCC CRL-2117) were maintained in Earl’s minimum essential medium (EMEM) (Invitrogen) supplemented with l-glutamine, MEM Non-Essential Amino Acids Solution, 100 U/ml penicillin, 100 µg/ml streptomycin and 10% fetal bovine serum. Flasks and 96-well plates were pre-coated with 0.1% gelatine (Millipore, Billerica, MA, USA) for adherence to surfaces. Cells were incubated in a humidified environment of 5% CO_2_ at 37 °C.

For the cell cytotoxicity assays, 96-well cell culture plates (BD Biosciences) were seeded with ~4 × 10^4^ LMH cells/well and incubated for 2–3 days until just 100% confluent. *C. perfringens* strains were grown in TPG broth to an OD_600nm_ of 0.6–0.8, and broth culture supernatants were obtained by centrifugation at 18,000×*g* for 15 min. The supernatants were filter-sterilized and added to the LMH cell medium (all samples were performed in triplicate), then incubated for 4 h at 37 °C with 5% CO_2_. Lactate dehydrogenase (LDH) release into the supernatant was measured as an indicator of cytolysis using the Cyto-Tox Non-Radioactive (Promega) kit. Triton-X 100 (1%) was used as positive control, which gave 100% cell death; the experiment was repeated three times.

### RT-PCR

Total RNA was isolated from overnight cultures of *C. perfringens* strains using RiboPure™-Bacteria Kit (Applied Biosystems) and DNase treated with the Ambion Turbo DNase kit (BioRad). All first-strand cDNAs were prepared from same amount of approximately 2.5 µg of total RNA in a 20 µl reaction containing 0.5 mM dNTPs, 1 µg random hexamers (Invitrogen), 1× First strand buffer, 10 mM dithiothreitol, 40 U RNasin (Promega) and 200 U Superscript II (Invitrogen). The reaction was incubated at 25 °C for 10 min, 42 °C for 50 min and 70 °C for 15 min. PCR amplification of transcribed *netB* was carried out using 2 µl of cDNA as template and primers QnetBF and QnetBR as described above.

### Infection of chickens

Fifty-four commercial 1-day-old male white Plymouth Rock broiler chickens (Bonnie’s Chick Hatchery, Elmira, ON, Canada) were randomly divided into 4 experimental groups (n = 13–14 per group). The chickens were fed an antibiotic-free chicken starter containing 20% protein for 11 days followed by turkey starter containing 28% protein (Arkell Research Station, University of Guelph). For the experimental infection (challenge) of birds, wild type CP1, CP1ΔpCP1netB and CP1ΔpCP1netB(*netB* ^+^) were grown in cooked meat medium (Difco) (CMM) for 24 h at 37 °C under anaerobic conditions. FTG medium (Difco) was then inoculated 3% (v/v) with an overnight CMM culture and incubated aerobically at 37 °C for 15 h. The growth at 15 h was approximately log_10_ 8.45 ± 0.14 *C. perfringens* colony-forming units (CFUs) per ml. The FTG-grown culture was then mixed with feed at a ratio of 2:1 (v/w). The chickens were starved for 24 h before the *C. perfringens* contaminated feed was administered on day 13. Inoculated feed was prepared fresh twice daily and fed to chickens for 5 days.

Chickens were euthanized with carbon dioxide on the day following a 5-day challenge, and at necropsy the small intestine was examined for grossly visible lesions. Necrotic enteritis lesions were scored blindly using the system described by Keyburn et al. [2].

To determine if there were differences in the number of birds with lesions between two challenge groups, a two-tailed Fisher’s exact test was used to assess the null hypothesis that the proportion of birds with lesions among the two groups was the same. The null hypothesis was rejected at p ≤ 0.05.

## Results

### Identification of a CP1 isolate deficient in pCP1netB

**Fig. 1:**
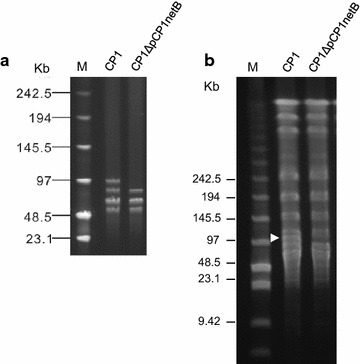
**a** PFGE profiles of large plasmids from CP1 and CP1ΔpCP1netB (digested with Not I). **b** Chromosomal SmaI-PFGE profiles of CP1 and CP1ΔpCP1netB. The *arrowhead* indicates the band missing in CP1ΔpCP1netB. *M* mid-range II PFG markers

Type A strain CP1 was chosen as the potential transformable host for transformation experiments. Of particular interest, after inoculation of a CP1 stock culture from −80 °C and several in vitro sub-cultures in BHI broth, we obtained one *netB* negative isolate which was also negative for several other genes located on NELoc-1 locus. This strain was named CP1ΔpCP1netB and its identification as a derivative of the virulent strain CP1 deficient in the plasmid (pCP1netB) that carries NELoc-1 was subsequently confirmed. Previously, our group reported that NELoc-1 is located on a ~85 kb plasmid in CP1, the largest of at least four plasmids carried by this strain. PFGE analysis of plasmid DNA from CP1 and CP1ΔpCP1netB confirmed that the pCP1netB had been lost. As expected, the plasmid PFGE profile of CP1ΔpCP1netB differed from the wildtype CP1 by the absence of the largest 85 kb plasmid band (Fig. 1a). This missing 85 kb band corresponded to the same size band previously shown, through Southern blotting experiments, to hybridize with a *netB* Dig-labelled probe. PFGE analysis of *Sma*I-digested chromosomal DNA from CP1 and CP1ΔpCP1netB revealed that the two strains otherwise share an identical PFGE pattern, with the exception of the absent band in the mutant that corresponds in size to pCP1netB (Fig. 1b).

**Fig. 2:**
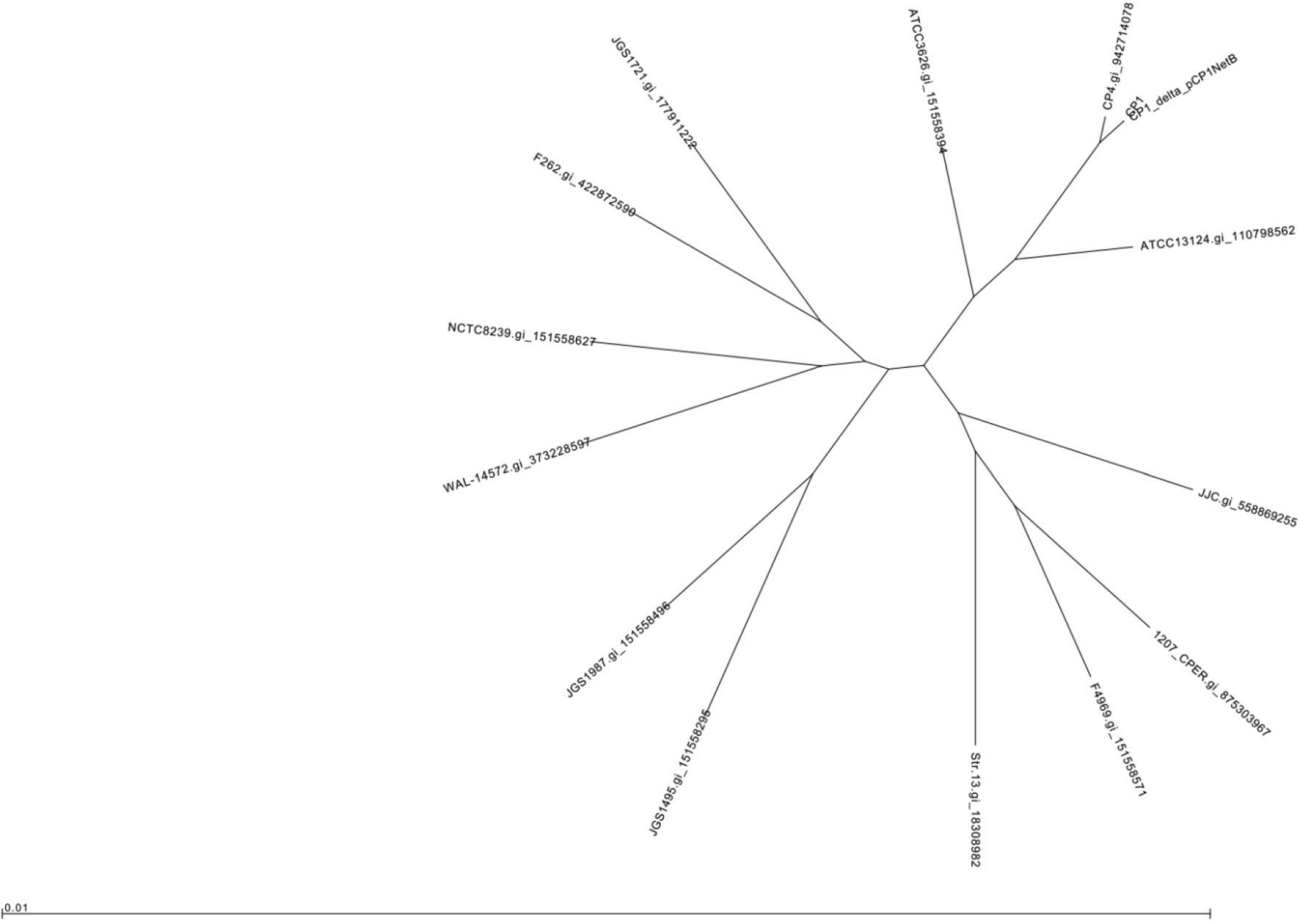
Phylogenetic tree based on whole genome alignments of CP1, CP1ΔpCP1netB generated with REALPHY. Whole genome sequences of CP1, CP1ΔpCP1netB and 12 additional *C. perfringens* genomes currently available in Genbank were aligned with REALPHY and a phylogenetic tree generated with RAxML. Strain names and gene accession numbers are given for each genome

To further confirm that CP1ΔpCP1netB was a derivative of CP1, genomic DNA from both isolates was sequenced using high throughput sequencing. Phylogenetic analysis of the two genomes, along with the genomes of 13 other *C. perfringens* strains currently available in GenBank, was performed using REALPHY, using ATCC 13124 as a reference genome. This tool reconstructs phylogeny based on single-nucleotide polymorphisms (SNPs) of conserved whole genome alignments, and is suitable for distinguishing closely related bacterial strains. As expected, CP1 and CP1ΔpCP1netB were indistinguishable from each other using this method, and were closely related to CP4, another poultry strain isolated from the same time and location as CP1 (Fig. 2). Other *C. perfringens* strains, though still closely related, were clearly separated into different branches on the phylogenetic tree. Mapping of the CP1ΔpCP1netB reads to the pNetB-NE10 sequence (JQ655731.1) also confirmed the absence of the NELoc1 pathogenicity locus (data not shown).

### Complementation of mutant CP1ΔpCP1netB with netB alone

**Fig. 3:**
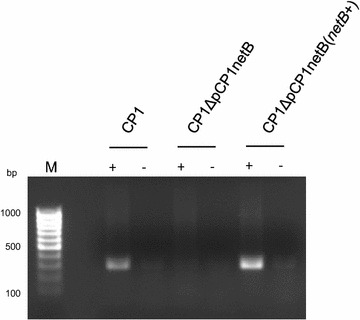
RT-PCR analyses for *netB *expression by CP1, CP1ΔpCP1netB and complementing strain CP1ΔpCP1netB(*netB* +). M, Low Range DNA Ladder; Lanes 1, 3, 5 (with +) were from samples receiving reverse transcriptase (RT), while lanes 2, 4, 6 (with −) lacked reverse transcriptase to show the absence of DNA contamination

In order to isolate the contribution of *netB* alone to the virulence of CP1, in the absence of other putative NELoc-1 virulence genes, a complemented strain CP1ΔpCP1netB(*netB* ^+^) was prepared by electroporating the mutant with pJIR750::*netB*, a pJIR750 derivative encoding the wildtype *C. perfringens netB* gene. When RT-PCR was performed to evaluate *netB* expression in these strains, both wildtype CP1 and the complemented strain supported amplification of a 302 bp *netB* product (Fig. 3). In contrast, no RT-PCR product was detected using template RNA isolated from the mutant. These results confirmed the expression of *netB* in the complemented strain.

### Cytotoxic effects towards LMH cells

**Fig. 4:**
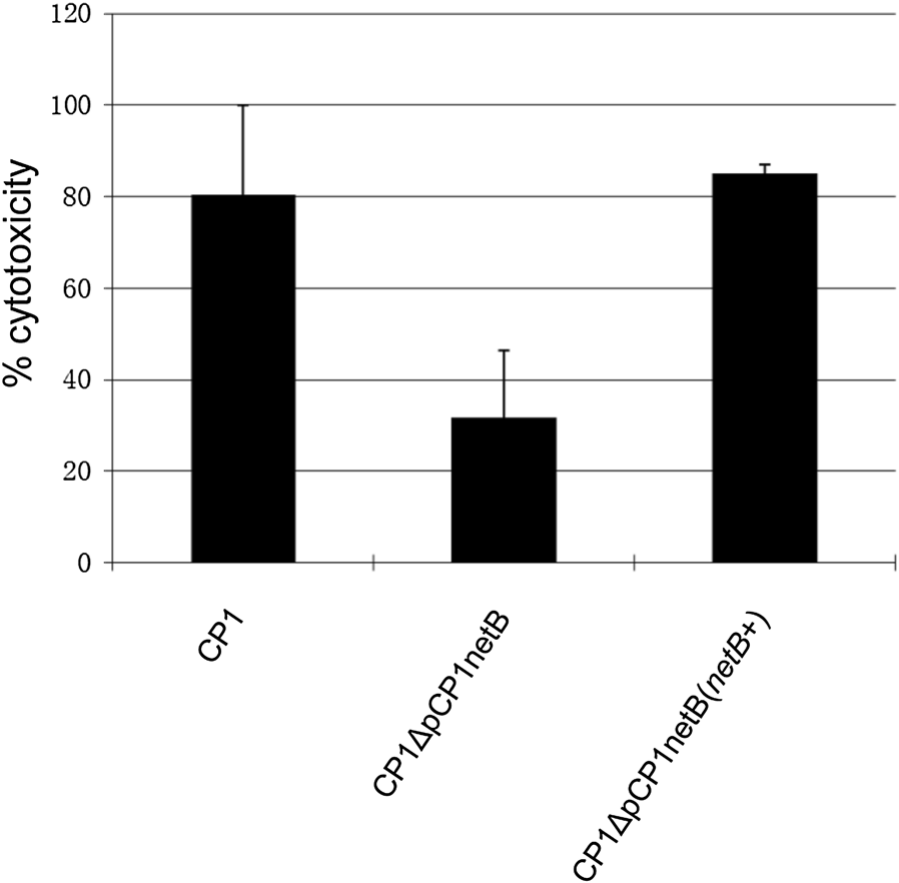
LMH cell cytotoxicity assay. Culture supernatants were isolated from CP1, CP1ΔpCP1netB and complementing strain CP1ΔpCP1netB(*netB* +). The experiment was repeated three times, and average results are shown. *Error bars* depict standard deviation (SD). The amount of cytotoxicity induced by NetB in each cell supernatant is expressed as a percentage

The NetB toxin has specific in vitro cytotoxic effects against the LMH cell-line. To quantitate the cytotoxicity of wildtype CP1, the mutant and the complemented strain, culture supernatants of these isolates were tested in the LMH cell cytotoxicity assay. The results showed that the level of cytotoxicity exhibited by culture supernatant derived from the mutant was significantly reduced compared to the wildtype control (Fig. 4). In contrast, complementation of the mutant with *netB* restored cytotoxicity levels to that found in CP1.

### Virulence of CP1ΔpCP1netB and CP1ΔpCP1netB(netB^+^) in vivo

**Fig. 5:**
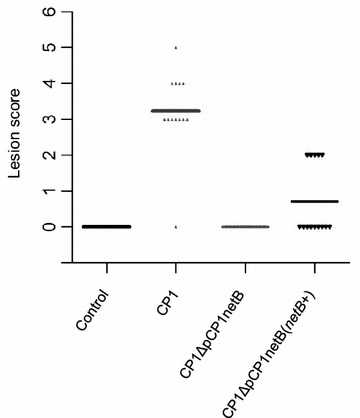
Lesion scores from chickens infected with different *C. perfringens* CP1 derivatives. The lesion scores of individual 18-day-old broiler chickens challenged with different *C. perfringens* strains are shown. Control group and CP1 group consisted of 13 birds; CP1ΔpCP1netB and complementing strain CP1ΔpCP1netB(*netB* +) groups consisted of 14 birds. The solid *horizontal bars* represent the average lesion score in each group

To determine the potential of *netB* alone to cause NE, four groups of broiler chickens were challenged with the wildtype, the mutant, and the complement of CP1 or FTG media alone in an NE infection model. No lesions were observed in birds fed with FTG medium (Control) or the mutant (Fig. 5). By contrast, significant levels of disease were detected in birds infected with the wildtype CP1, with an average lesion score of 3.23. In the group infected with the complemented pNetB-negative isolate, five out of 14 birds had lesion score of 2, generating an average lesion score of 0.71. The proportion of infected birds in the complemented group was significantly less (Fisher’s exact test, p ≤ 0.05) than that of the wildtype CP1 group.

## Discussion

In this study, we have characterized a *C. perfringens* mutant from a chicken NE strain (CP1) that spontaneously lost the plasmid carrying the NE-specific locus NELoc-1, including investigating its cytotoxicity towards the chicken male Leghorn hepatoma cell-line and capacity to produce NE lesions in broilers. The validity of this CP1 mutant (CP1ΔpCP1netB) as lacking only the pNetB plasmid and having no other gene changes was confirmed by PFGE of plasmid and chromosomal DNA and genome sequencing (Figs. 1, 2).

Previously, our group reported on three highly conserved NE-associated loci that were designated NELoc-1 (42 kb), NELoc-2 (11.2 kb) and NELoc-3 (5.6 kb). The largest locus, NELoc-1, consists of *netB* and 36 additional genes [7]. In the present study, we tested the virulence conferred by *netB* alone, in the absence of the other NELoc-1 genes. The expression of *netB* was sufficient to restore the in vitro cytotoxicity of CP1ΔpCP1netB to wildtype levels, as observed when supernatant from the complemented CP1ΔpCP1netB(*netB* ^+^) was tested in the LMH cytotoxicity assay (Fig. 4). Cytotoxicity of *C. perfringens* culture supernatants towards the LMH cell-line has been demonstrated to be specifically attributable to NetB [17], and has been used extensively to assay the activity of NetB [2, 15, 18]. Our study importantly suggests that genes on the pNetB pathogenicity island (other than *netB*) are not involved directly in regulating expression of NetB.

When assessed in vivo, the complemented group failed to fully restore the ability of CP1ΔpCP1netB to cause NE (Fig. 5). While it has been reported that there is not always a direct correlation between in vitro cytotoxicity and the development of NE [19], the inability of *netB* complementation alone to restore full virulence to CP1ΔpCP1netB also suggests that other genes present on pCP1netB are required during NE pathogenesis in a role not related to NetB expression. NELoc-1 encodes a number of genes with putative virulence-related functions, including adhesins, glycosidases, and a cyclic-di-GMP signaling system [7], which plausibly influence the survival and colonization of *C. perfringens* in the intestinal mucosa during NE pathogenesis, as well as other possible functions. The fact that neither *netB* transcription determined through semi-quantitative RT-PCR, nor in vitro LMH cytotoxicity appeared to be reduced by CP1ΔpCP1netB(*netB* ^+^), indicates that this isolate has the capacity similar to the wild-type strain in the production of NetB toxin. In addition, the uneven distribution of NE disease (approximately one-third of the birds showed NE disease but two-thirds had no NE lesion) and less severe NE lesions in the complemented group make it difficult to attribute the results to the loss of the vector, given that all the birds received the same delivery of the isolate through a diet during the challenge. Thus, our results support the hypothesis that although NetB is the essential factor which initiates NE disease, its delivery to target cells clearly involves a complex delivery system likely including mucus and mucosal adhesion [21].

It should be noted that the disease model system employed in this study, and by others, may be insufficient for assessing the contribution to pathogenesis of all accessory virulence factors, which could each play a role at any point during the infection cycle of the pathogen [20, 21]. For example, genes involved in environmental survival, transmission, competition with the indigenous intestinal microbiota, or other activities that may normally be essential for pathogenesis, may no longer be required due to the severe challenge used in this model system. Hence, more sensitive in vivo assays directly suited to evaluate these functions need to be explored.

## Conclusions

The current study found that *netB*, in the absence of other NELoc-1 genes, was able to completely restore in vitro cytotoxicity, but only partially restore virulence in vivo. These results importantly support the hypothesis that other genes present on NELoc-1 are involved in NE pathogenesis and have functions other than controlling the expression of NetB.
